# The value of robot-assisted gastrectomy in the treatment of gastric cancer: a systematic review and meta-analysis

**DOI:** 10.3389/fsurg.2026.1828368

**Published:** 2026-06-09

**Authors:** Lei Chen, Qian Wang, Wanbin He, Yonghong Wang, Yaping Li, Jie Dan

**Affiliations:** 1 Department of Gastrointestinal Surgery, People's Hospital of Leshan, Leshan, Sichuan, China; 2 Department of Ultrasonic Medicine, People's Hospital of Leshan, Leshan, Sichuan, China

**Keywords:** gastric cancer, meta-analysis, oncological outcomes, open gastrectomy, robotic gastrectomy

## Abstract

**Background:**

Open gastrectomy (OG) has long been the standard for gastric cancer surgery. Robotic gastrectomy (RG) has emerged as a minimally invasive alternative with potential technical advantages, but large-sample meta-analyses comparing their clinical value are limited. This study aims to evaluate its clinical practical application value and provide a reference for clinical practice.

**Methods:**

A systematic search was conducted in PubMed, EMBASE, MEDLINE, Web of Science, and Cochrane Library up to July 2025, following PRISMA guidelines, with statistical analysis using STATA 12.

**Results:**

Seventeen studies involving 31,573 patients (10,524 RG, 21,049 OG) were included. RG had longer operative time (WMD: 93.4; 95% CI: 65.89–120.9; *P* < 0.001) but less blood loss (WMD: −86.71; 95% CI: −120.91–−52.52; *P* < 0.001), shorter hospital stay (WMD: −3.17 days; 95% CI: −4.17–−2.16; *P* < 0.001), lower postoperative complication rate (OR: 0.61; 95% CI: 0.43–0.87; *P* = 0.006), and higher R0 resection rate (OR: 1.88; 95% CI: 1.57–2.24; *P* = 0.000). No significant differences were found in lymph nodes, positive lymph nodes, postoperative mortality, or 5-year survival.

**Conclusion:**

RG is a reasonable choice for proficient robotic surgery centers, balancing invasiveness and oncological precision. Future studies should focus on long-term outcome and quality of life to guide clinical decision-making.

## 1 Introduction

Gastric cancer (GC) is a prevalent malignancy of the digestive system, ranking as the third leading cause of cancer-related deaths globally, following lung, colorectal, and liver cancer, according to the International Agency for Research on Cancer's Global Cancer Observatory (GLOBOCAN) (1). Notably, this disease is increasingly affecting younger individuals. The primary therapeutic approach is surgical intervention, with open gastrectomy (OG) historically considered the standard procedure. However, the emergence of minimally invasive techniques, such as laparoscopic gastrectomy (LG) and robot-assisted gastrectomy (RG), has gained popularity due to their reduced invasiveness, faster patient recovery, and decreased intraoperative bleeding (2, 3). Robotic-assisted surgical systems equipped with high-definition three-dimensional visualization, scaling, and ergonomic tremor cancellation offer the potential to overcome the limitations of conventional laparoscopy, particularly in skill-intensive procedures such as lymph node dissection and gastrointestinal anastomosis creation (4).

However, the high costs, steep learning curve, and uncertain oncological outcomes have raised concerns about their widespread clinical applicability (5). While some studies have suggested that LG) outperforms OG in terms of intraoperative blood loss, postoperative complications, and hospital stay (6–8). Nevertheless, there is a scarcity of studies that directly compare the oncological outcomes between RG and OG. Therefore, a method that is more beneficial or more useful than OG for gastrectomy in patients with GC has yet to be determined. Currently, no large-sample meta-analysis has been performed to compare the efficacy and safety of RG versus OG for GC, Especially the lack of evaluation of long-term outcomes. Therefore, given the lack of evidence, this study aims to evaluate the value of RG in the treatment of GC through a large-sample meta-analysis, providing evidence for clinical decision-making.

## 2 Materials and methods

### 2.1 Literature retrieval

This review adheres to the PRISMA guidelines (9). The research protocol for this meta-analysis was preregistered in the Cochrane Systematic Review Register under registration number CRD420251140403. Three authors systematically searched PubMed, EMBASE, MEDLINE, Web of Science, and the Cochrane Central Register of Controlled Trials for controlled studies comparing RG and OG for GC, with a specific emphasis on comprehensive outcome reporting. The literature search was limited to English studies published until July 2025. Any disagreements were addressed by a fourth reviewer. The search terms included “gastric cancer” “robotic gastrectomy” “open gastrectomy” and “gastrectomy”. A manual search supplemented the database search, and reference lists of included studies were examined for further information.

### 2.2 Inclusion and exclusion criteria

Inclusion criteria included: 1) Patients diagnosed with gastric cancer through imaging or pathology; 2) Surgical gastrectomy with lymph node dissection performed; 3) Randomized controlled trials (RCTs), retrospective cohort study (RCS), or comparative studies, irrespective of patient demographics; 4) No preoperative treatments such as surgery, radiotherapy, or neoadjuvant chemotherapy; 5) Comparison of postoperative outcomes, including at least one of the following: operative time, blood loss, hospital stay, lymph nodes, positive nodes, R0 resection, postoperative complication, postoperative mortality, or 5-year survival rate; 6) Availability of full-text articles.

Exclusion criteria included: 1) Studies not aligning with inclusion criteria; 2) Research subjects who underwent gastric cancer surgery due to other diseases or recurrent gastric cancer; 3) Abstracts without full texts or sufficient information; 4) Non-English articles; and 5) Advanced indications unrelated to gastrectomy.

### 2.3 Literature screening and data extraction

Three authors independently reviewed the full text of each study to extract key data, including the first author, publication year, study design, surgical procedure, sample size, and outcomes. All propensity score matching (PSM) data were reported post-matching. For quantitative data lacking mean and standard deviation (SD), we estimated these values using alternative methods (10, 11) when the authors did not provide the missing information, utilizing the median, interquartile range (IQR), range, and sample size. For studies that did not directly report the hazard ratio (HR) and its 95% confidence interval (CI), we extracted survival probabilities from Kaplan–Meier curves using Engauge Digitizer software and employed the Parmar method (12) to convert the chi-square value from the log-rank test *P*-value and estimate the HR and 95% CI.

### 2.4 Methodological quality assessment

The quality of non-RCT studies was assessed using the Newcastle–Ottawa Scale (NOS) (13), which has been widely used in the quality evaluation of nonrandomized studies. This scale was used to rate the quality of the included studies, based on the selection of the study population, comparability of the groups under study, and outcome assessment. The maximum score on the scale was 9, with scores > 5 indicating high methodological quality. Any disagreements in scoring were resolved through consensus.

### 2.5 Data statistical analysis

Data analysis was conducted using STATA 12. For binary variables, odds ratios (OR) with 95% CI were reported. Continuous variables were assessed using the weighted mean difference (WMD) and 95% CI, and a pooled effect size was calculated for each study. Statistical significance was defined as (*P* < 0.05). The I² statistic was used to assess heterogeneity between effect estimates, with I^2^ > 50% indicating substantial statistical heterogeneity. If heterogeneity was considered negligible, a fixed-effect model was used. If heterogeneity was present (> 50%), the sources of heterogeneity were first analyzed. If there was no apparent clinical heterogeneity, a random-effects model was used, and conduct subgroup analysis based on geographic region. Any significant clinical heterogeneity was addressed using sensitivity analysis, Sensitivity analysis was conducted by removing individual studies and repeating the meta-analysis. Publication bias was assessed with a funnel plot, and its asymmetry tested using Begg's and Egger's tests. Results were deemed statistically significant at *P* < 0.05, with Egger's test results taking precedence in case of inconsistency.

## 3 Results

### 3.1 Literature retrieval, characteristics, and quality

**Figure 1:**
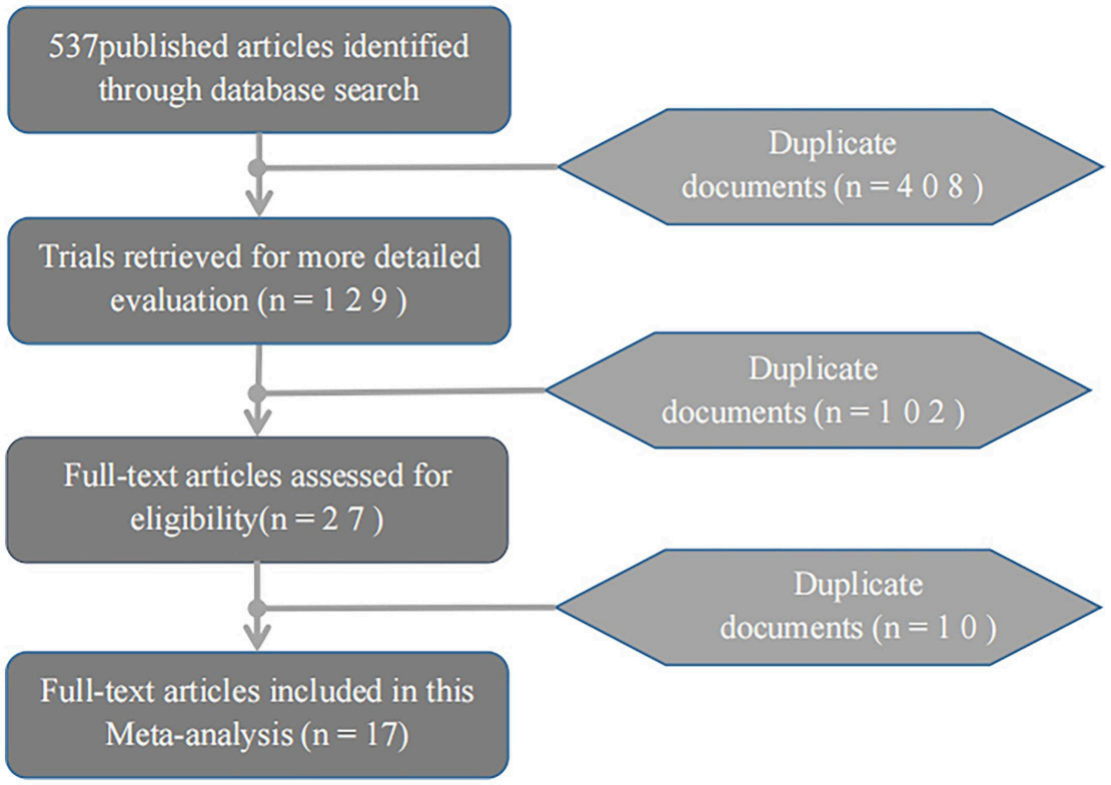
Flow diagram of literature selection.

A total of 537 articles were initially identified using the specified retrieval method, with 408 duplicates removed. After screening titles and abstracts, 102 studies were selected for full-text review. Based on the inclusion and exclusion criteria and data completeness, a total of 17 studies were included (6, 14–29), involving 31,573 patients, with 10,524 in the RG group and 21,049 in the OG group. The literature screening process is shown in Figure 1. All included studies were RCS, so the NOS scale was used for quality assessment, and the basic characteristics and quality scores of the included studies are detailed in Table 1.

**Table 1:** Characteristics and quality scores of included studies.

Studies	Year	Country	Study Design	Age( years)^a^	Sample Size			Outcomes	NOS
					Total	RG	OG		
Kim et al. (14)	2010	Korea	RCS	53.80 ± 15.60/ 56.00 ± 12.40	28	16	12	①②③⑦	7
Caruso et al. (15)	2011	Italy	RCS	64.80 ± 12.40/ 65.10 ± 11.00	149	29	120	①②③④⑤⑥⑦⑧	7
Huang et al. (16)	2012	China	RCS	65.10 ± 15.90/ 67.90 ± 30.10	625	39	586	⑦⑧④	6
Kim et al. (17)	2012	Korea	RCS	54.20 ± 12.50/ 57.70 ± 11.80	4978	436	4542	①②③④⑤⑥⑦	6
Procopiuc et al. (18)	2016	Romania	RCS	59.17 ± 13.70/ 60.17 ± 12.50	47	18	29	①②③④⑦	7
Parisi et al. (19)	2017	Italy	RCS	68.81 ± 12.12/ 67.19 ± 13.1	453	151	302	①②③④⑥⑦	6
Yang et al. (20)	2017	Korea	RCS	-	416	173	243	①②③④⑥⑦⑧	7
Caruso et al. (21)	2018	Spain	RCS	62.00 ± 14.50/ 68.92 ± 6.80	39	20	19	①②③④⑦⑧	6
Solaini et al. (22)	2019	Italy	RCS	-	98	49	49	①③④⑤⑥⑦⑧	5
Caruso et al. (6)	2020	Spain	RCS	64.00 ± 12.50/ 68.78 ± 7.80	50	25	25	①②③④⑥⑦⑧	7
Garbarino et al. (23)	2020	Italy	RCS	77.50 ± 4.20/ 78.50 ± 5.30	86	43	43	①②③④⑤⑥⑦⑧⑨	6
Watson et al. (24)	2020	America O	RCS	65.00 ± 11.85/ 66.00 ± 12.59	7849	7161	688	④	7
Trastulli et al. (28)	2023	Germany	RCS	68.70 ± 12.20/ 68.10 ± 11.90	580	290	290	①②③④⑤⑥⑦⑧⑨	7
Hirata et al. (25)	2023	USA	PCS	60.00 ± 10.50/ 64.00 ± 8.83	161	41	120	①②③④⑥	7
Hirata et al. (26)	2023	USA	RCS	65.00 ± 13.16/ 67.00 ± 12.59	16465	1867	14598	③④⑥	7
Salvador et al. (27)	2023	Spain	RCS	68.00 ± 13.00/ 64.00 ± 13.00	78	30	48	①③④⑤⑥⑦⑧	6
Yaman et al. (29)	2025	Turkey	RCS	63.30 ± 9.80/ 63.90 ± 12.80	93	36	57	①②③④⑤⑥⑦⑧	7

RG, Robotic gastrectomy; OG, Open gastrectomy; -, Not available; NOS, Newcastlee-Ottawa Scale.

Outcomes: ①operative time, ②blood loss, ③hospital stay, ④lymph nodes, ⑤positive lymph nodes, ⑥R0 resection, ⑦postoperative complication, ⑧postoperative mortality, ⑨5-year survival rate.

a RG group/OG group

### 3.2 Meta-analysis of operative time (min)

**Figure 2:**
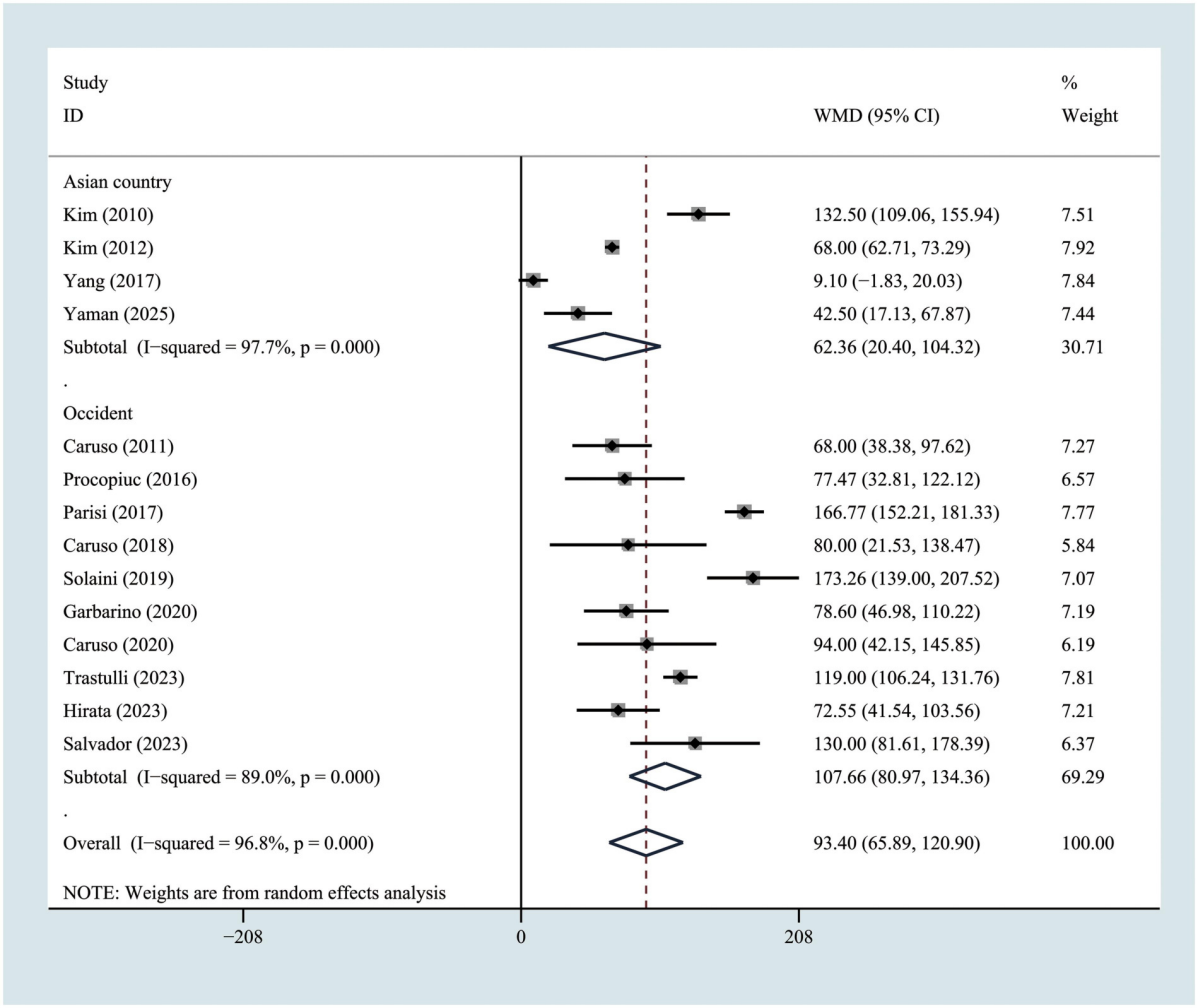
The forest plot of the total and subgroup analysis for operative time.

Fourteen studies (6, 14, 15, 17–23, 25, 27–29) provided clear data on operative time. Significant heterogeneity among the studies was observed (I^2^ = 96.8%, *P* = 0.000), and the random-effect model was used for analysis. The total combined analysis showed that the operative time was significantly prolonged in the RG in comparison to the OG, and the difference was statistically significant (WMD: 93.4; 95% CI: 65.89–120.9; *P* < 0.001). In subgroup analysis, four Asian country and ten occident all showed similar overall results, while the different results were statistically significant, *P* < 0.001, Figure 2. The sensitivity analysis verified the stability of the results.

### 3.3 Meta-analysis of blood loss (mL)

**Figure 3:**
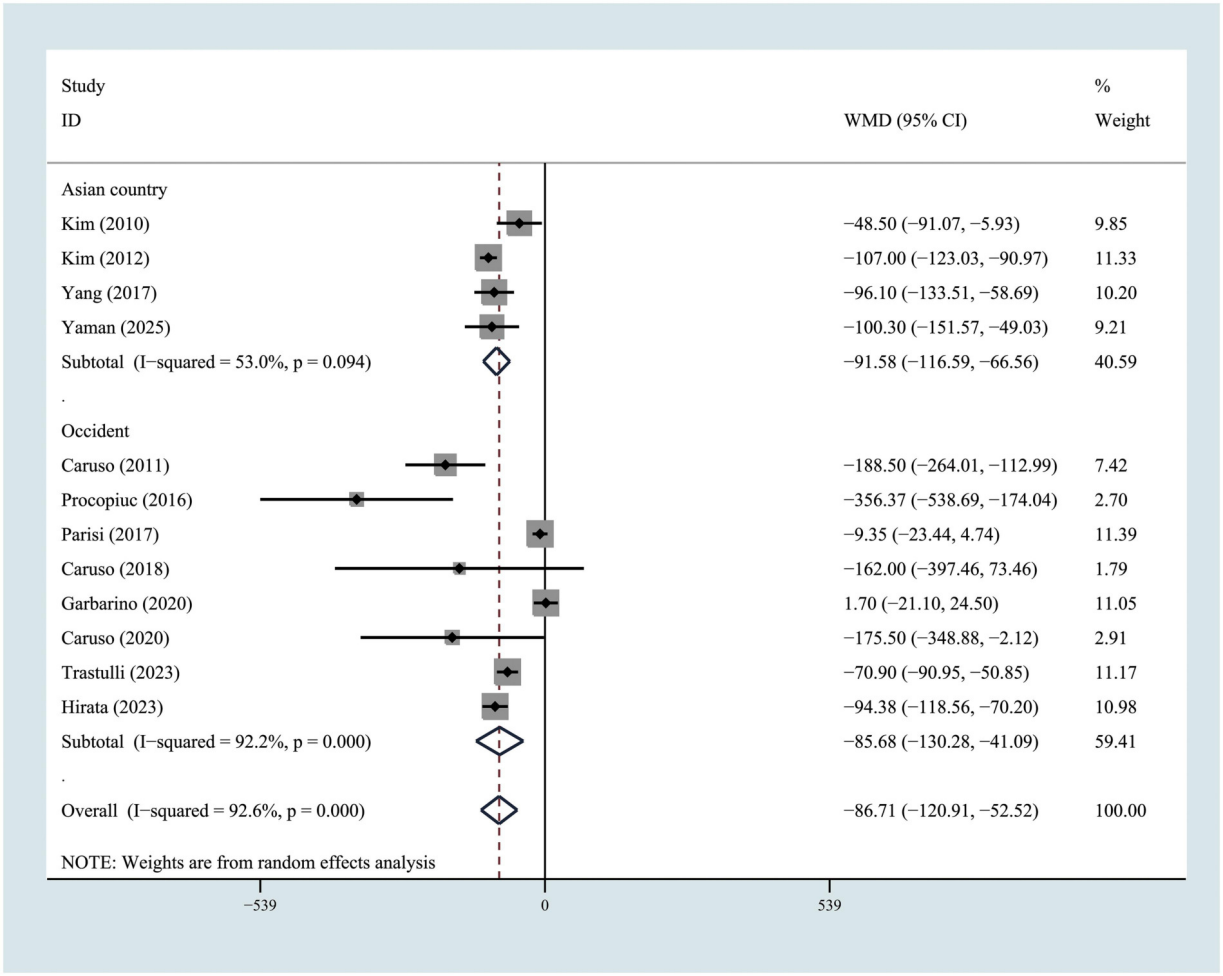
The forest plot of the total and subgroup analysis for blood loss.

Twelve studies (6, 14, 15, 17–22, 25, 28, 29) provided clear data on blood loss. Significant heterogeneity among the studies was observed (I^2^ = 92.6%, *P* = 0.000), and the random-effect model was used for analysis. The total combined analysis showed that the blood loss was significantly lower in the RG group compared to the OG group, and the difference was statistically significant (WMD: −86.71 mL; 95% CI: −120.91–−52.52; *P* < 0.001). In subgroup analysis, four Asian country and eight occident all showed similar overall results, while the different results were statistically significant, *P* < 0.001, Figure 3. The sensitivity analysis verified the stability of the results.

### 3.4 Meta-analysis of hospital stay (day)

**Figure 4:**
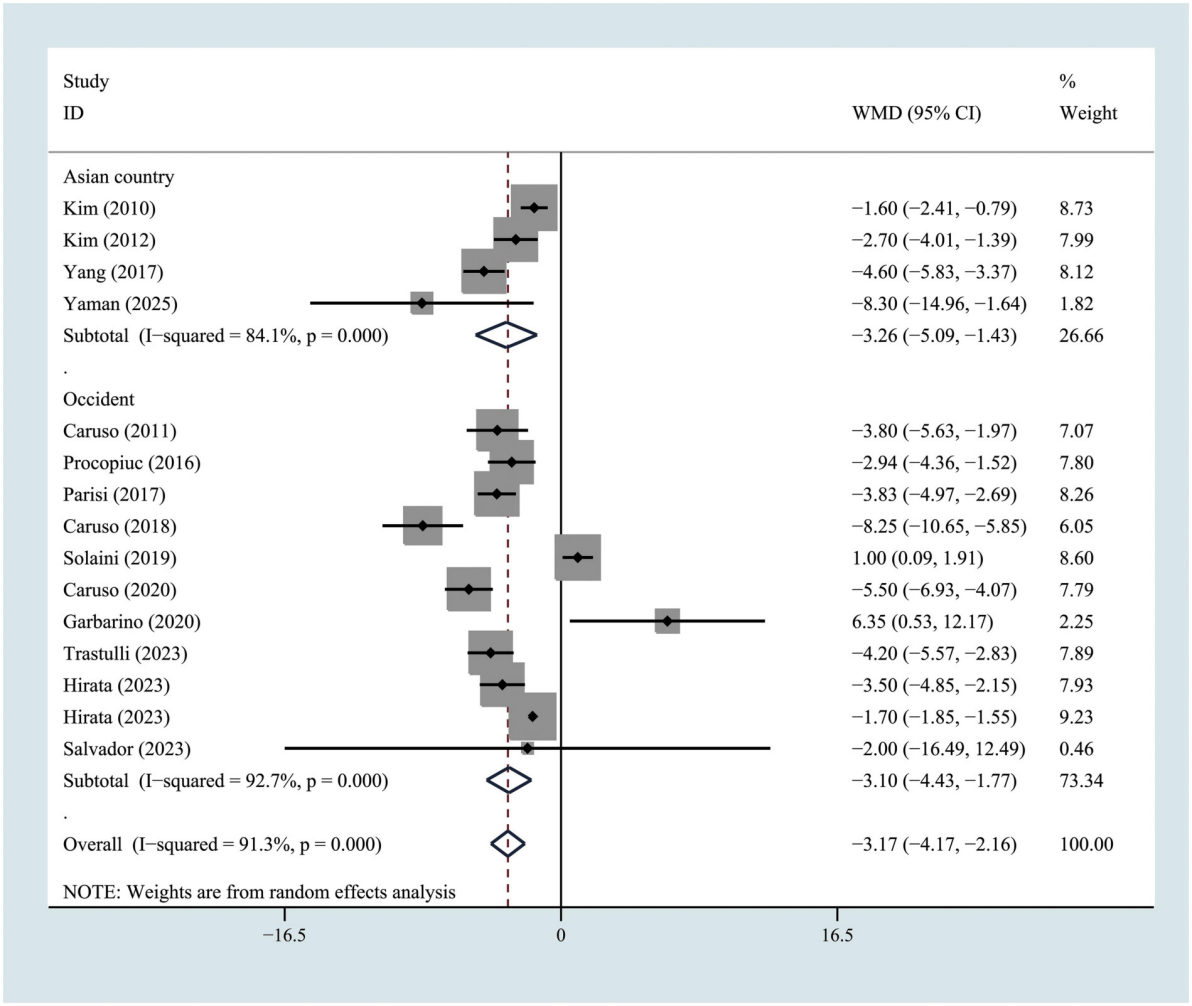
The forest plot of the total and subgroup analysis for hospital stay.

Fifteen studies (6, 14, 15, 17–23, 25–29) provided clear data on hospital stay. Significant heterogeneity among the studies was observed (I^2^ = 91.3%, *P* = 0.000), and the random-effect model was used for analysis. The total combined analysis showed that the hospital stay was significantly reduced in the RG group compared to the OG group, and the difference was statistically significant (WMD: −3.17 days; 95% CI: −4.17–−2.16; *P* < 0.001). In subgroup analysis, four Asian country and eleven occident all showed similar overall results, while the different results were statistically significant, *P* < 0.001, Figure 4. The sensitivity analysis verified the stability of the results.

### 3.5 Meta-analysis of lymph nodes

**Figure 5:**
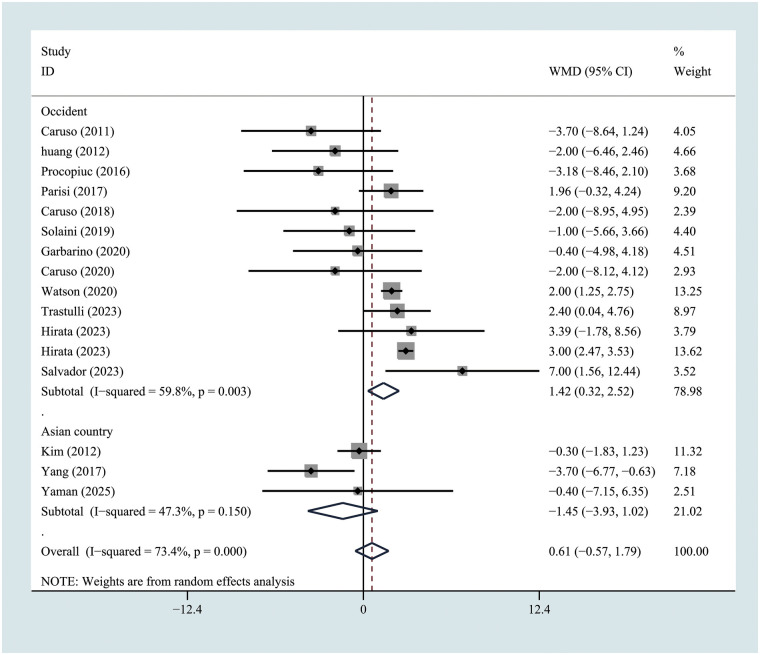
The forest plot of the total and subgroup analysis for lymph nodes.

Sixteen studies (6, 15–29) provided clear data on lymph nodes. Significant heterogeneity among the studies was observed (I^2^ = 73.4%, *P* = 0.000), and the random-effect model was used for analysis. The total combined analysis showed that no statistically significant in lymph node between the RG and OG groups. In subgroup analysis, tree asian country also showed similar overall results. But, thirteen occident showed RG has more lymph nodes than OG, and the difference was statistically significant (WMD: 1.42; 95% CI: 0.32–2.52; *P* < 0.05), Figure 5. The sensitivity analysis verified the stability of the results.

### 3.6 Meta-analysis of positive lymph nodes

**Figure 6:**
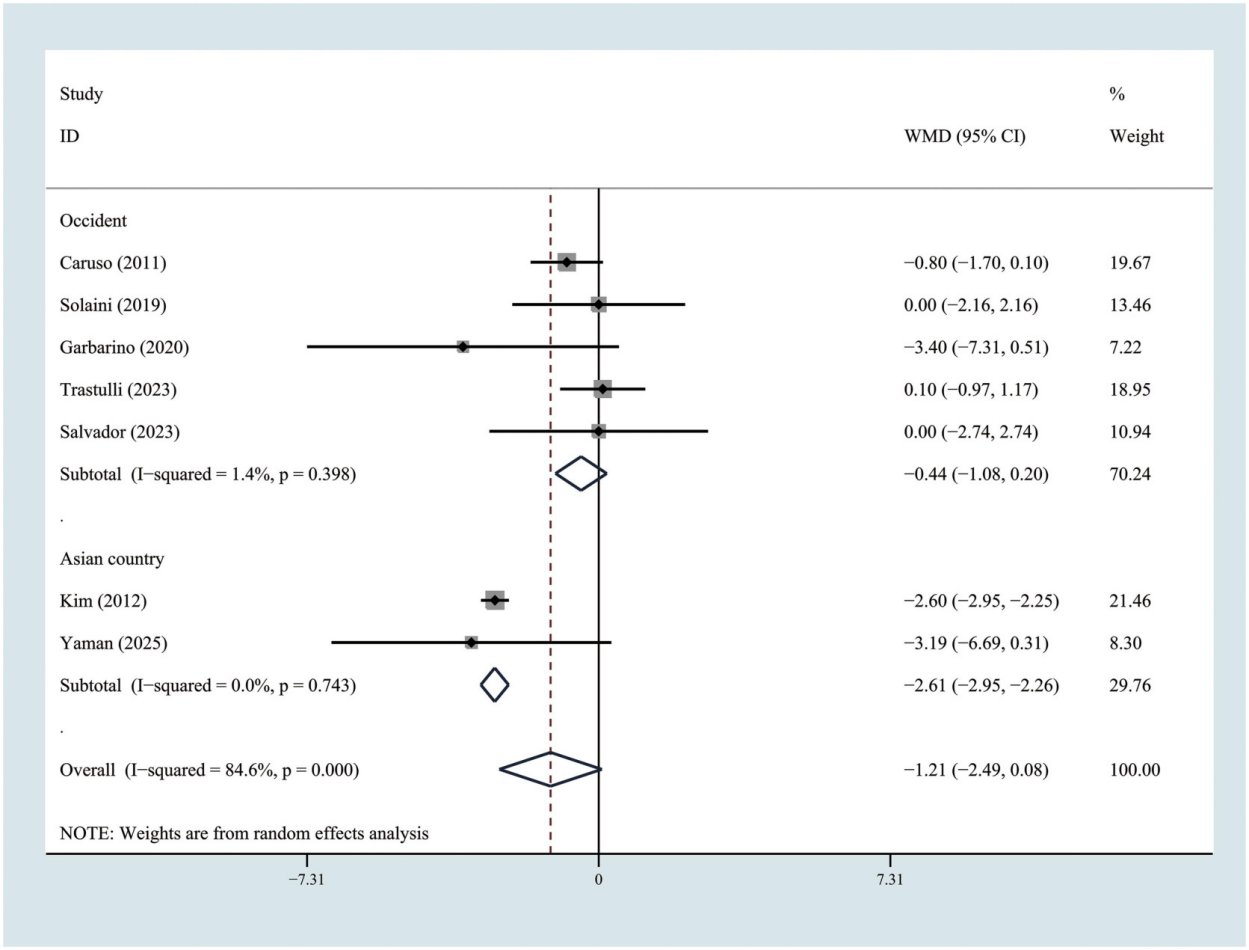
The Forest plot of the total and subgroup analysis for positive lymph nodes.

Seven studies (15, 17, 22, 23, 27–29) provided clear data on positive lymph nodes. Significant heterogeneity among the studies was observed (I^2^ = 84.6%, *P* = 0.000), and the random-effect model was used for analysis. The total combined analysis showed that no statistically significant inpositive lymph nodes between the RG and OG groups. In subgroup analysis, five occident also showed similar overall results. But, two asian country showed RG has lesser positive lymph nodes than OG, and the difference was statistically significant (WMD: −2.61; 95% CI: −2.95–−2.26; *P* < 0.001), Figure 6. The sensitivity analysis verified the stability of the results.

### 3.7 Meta-analysis of R0 resection

**Figure 7:**
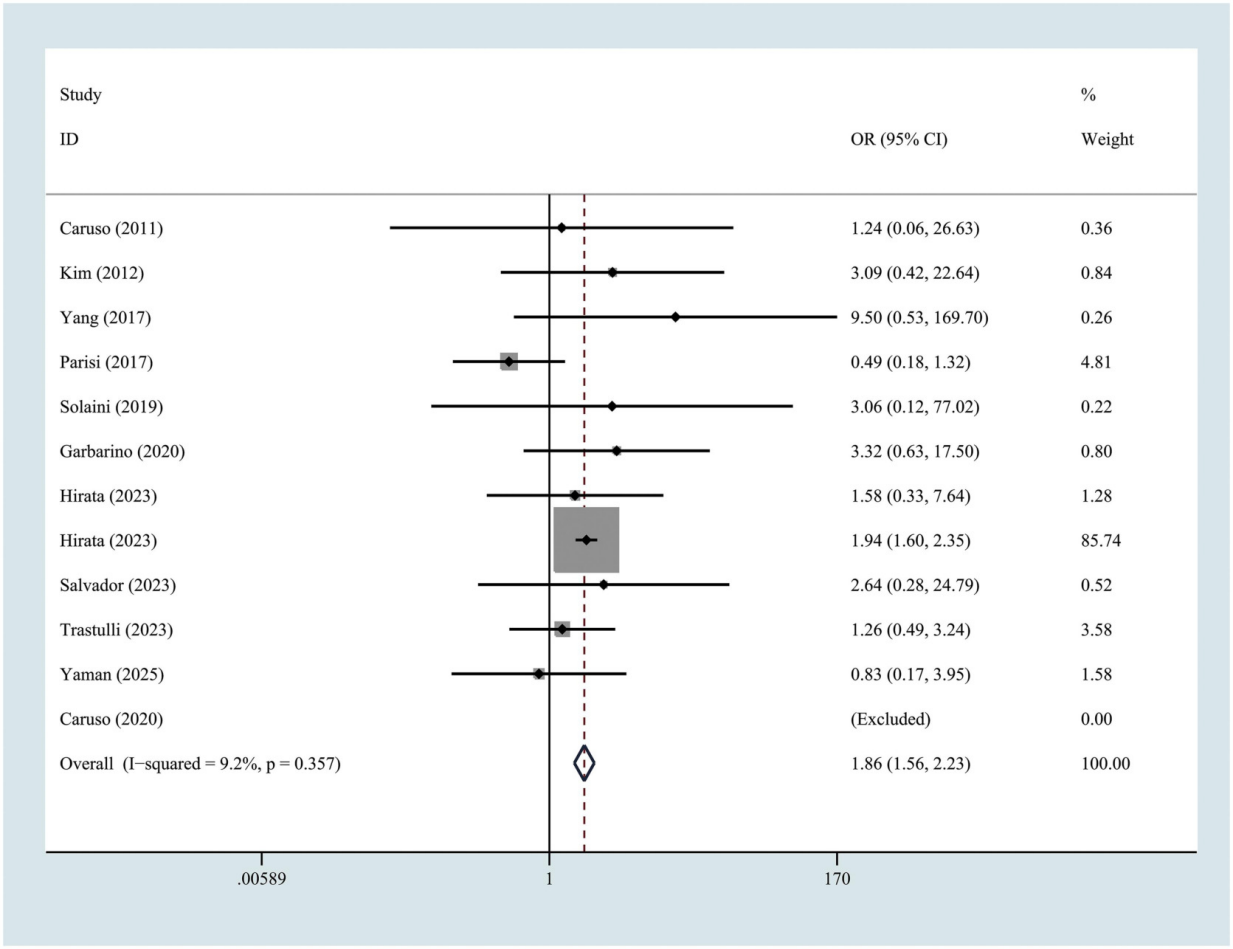
The forest plot of the R0 resection.

Twelve studies (6, 15, 17, 19, 20, 22, 23, 25–29) provided clear data on R0 resection. No heterogeneity among the studies was observed (I^2^ = 9.2%, *P* = 0.357), and the fixed-effects model was used for analysis. The total combined analysis showed that R0 resection was significantly higher in the RG group compared to the OG group, and the difference was statistically significant (OR: 1.86; 95% CI: 1.56–2.23; *P* = 0.000) (Figure 7).

### 3.8 Meta-analysis of postoperative complication

**Figure 8:**
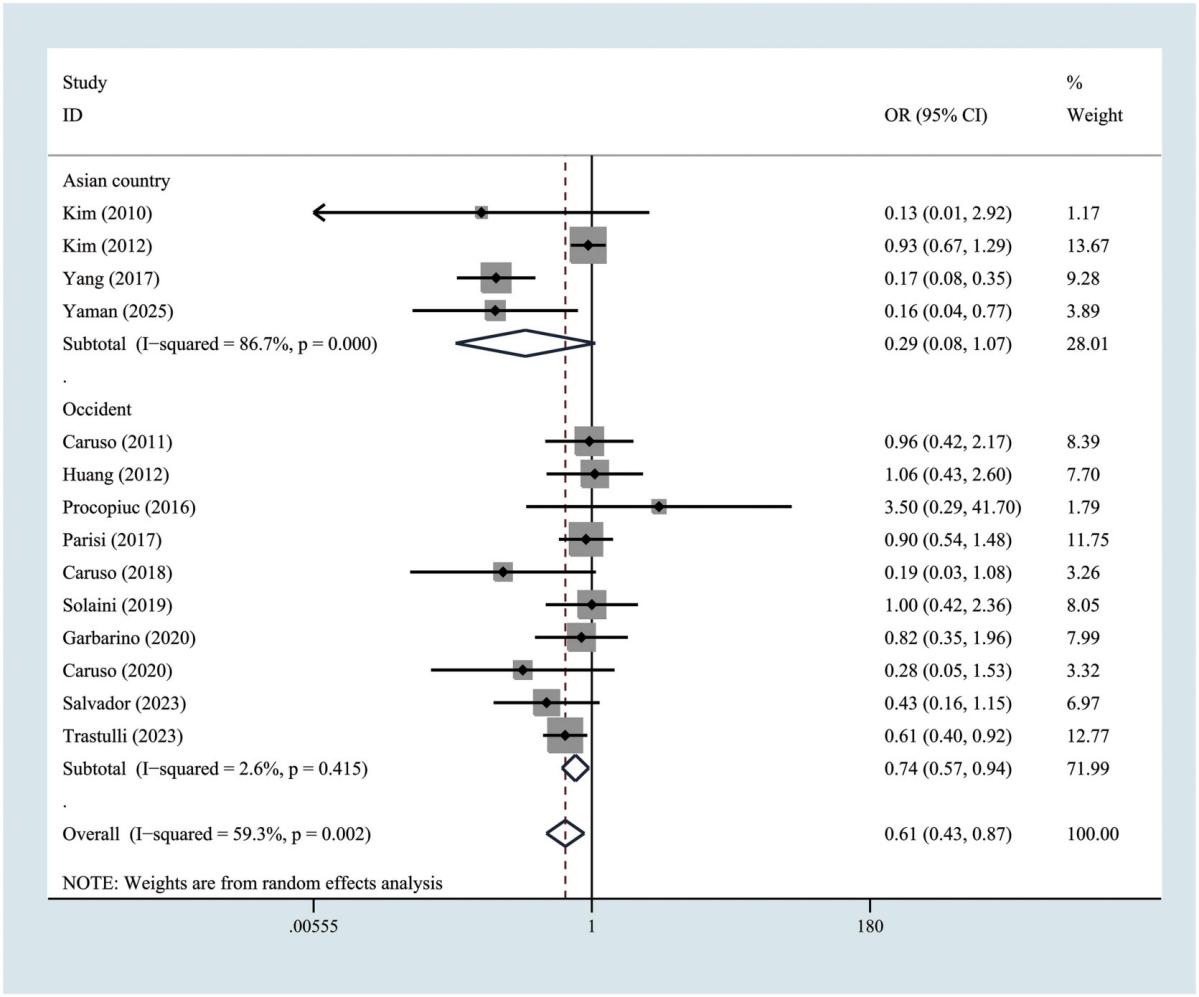
The forest plot of the total and subgroup analysis for postoperative complication.

Fourteen studies (6, 14–23, 27–29) provided clear data on postoperative complication. Significant heterogeneity among the studies was observed (I^2^ = 59.3%, *P* = 0.002), and the random-effect model was used for analysis. The total combined analysis showed that the postoperative complication was significantly lower in the RG group compared to the OG group, and the difference was statistically significant (OR: 0.61; 95% CI: 0.43–0.87; *P* = 0.006). In subgroup analysis, ten occident also showed similar overall results. But, four asian country showed that no statistically significant in postoperative complication between the RG and OG groups, Figure 8. The sensitivity analysis verified the stability of the results.

### 3.9 Meta-analysis of postoperative mortality

**Figure 9:**
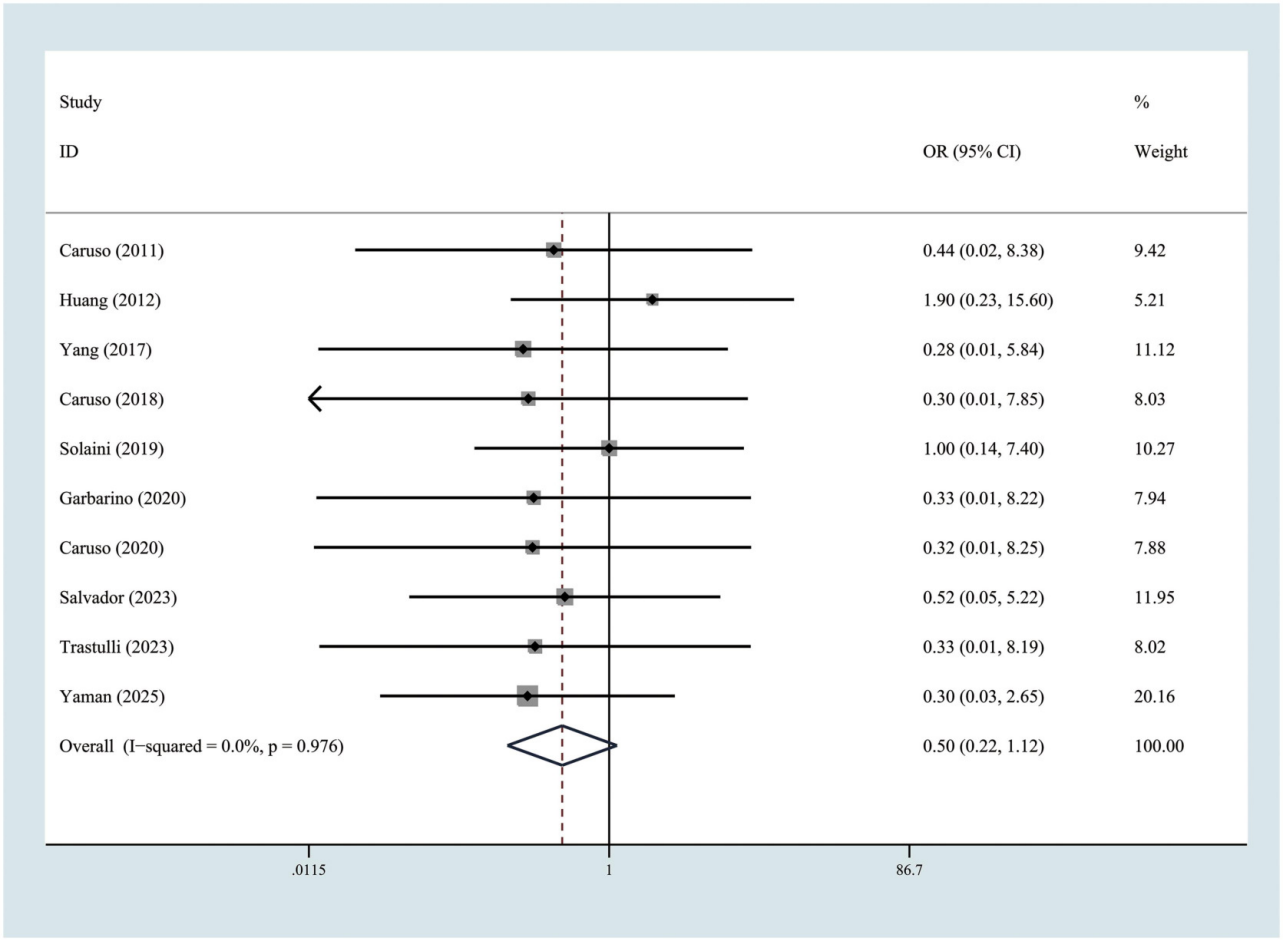
The forest plot of the postoperative mortality.

Ten studies (6, 15, 16, 20–23, 27–29) provided clear data on postoperative mortality. No heterogeneity among the studies was observed (I^2^ = 0.0%, *P* = 0.976), and the fixed-effects model was used for analysis. The total combined analysis showed that no statistically significant in postoperative mortality between the RG and OG groups (OR: 0.50; 95% CI: 0.22–1.12; *P* = 0.093) (Figure 9).

### 3.10 Meta-analysis of 5-year survival rate

**Figure 10:**
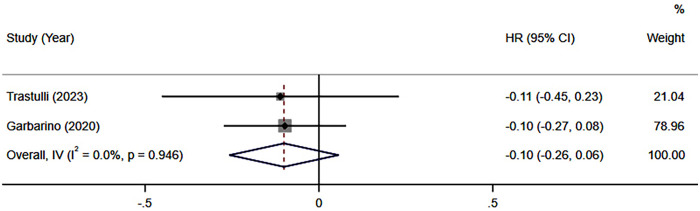
The forest plot of the 5-year survival rate.

Two studies (23, 28) provided clear data on 5-year survival rate. No heterogeneity among the studies was observed (I^2^ = 0.0%, *P* = 0.947), and the fixed-effects model was used for analysis. The analysis revealed no statistically significant in 5-year survival rate between the RG and OG groups (HR: −0.1; 95% CI: −0.26–0.06; *P* = 0.207) (Figure 10).

### 3.11 Publication bias and sensitivity analysis

**Figure 11:**
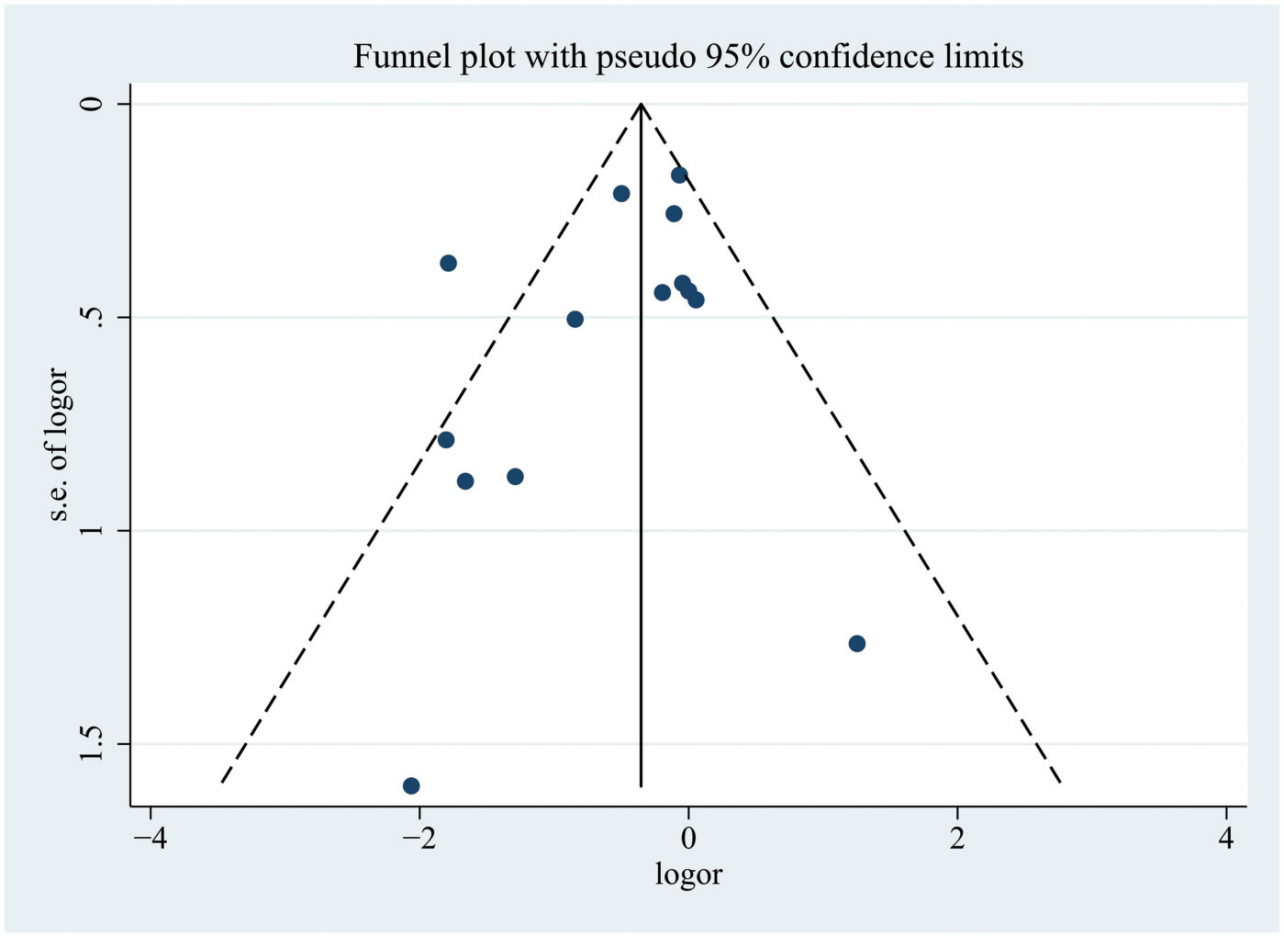
Publication bias funnel plot based on postoperative complications.

Heterogeneity results showed significant heterogeneity for operation times, blood loss, hospital day, lymph nodes, positive lymph nodes and postoperative complication. Sensitivity analyses were conducted by sequentially excluding individual studies. The results showed that no single study was found to significantly affect the stability of the above-mentioned outcome indicators (Supplementary Figures S1–S6). Publication bias was assessed using a funnel plot based on postoperative complication (Figure 11). Begg's and Egger's tests were conducted to detect funnel plot asymmetry, with significant publication bias indicated by *P* < 0.05. The symmetrical funnel plot suggested the absence of notable publication bias in the analysis (Begg's test: *P* = 0.228; Egger's test: *P* = 0.182).

## 4 Discussion

The treatment approach for gastric cancer has evolved, with current emphasis on individualized multimodal combination therapies resulting in notable enhancements in both oncological and survival outcomes (30). Surgeons worldwide are now striving to minimize surgical trauma and postoperative complications while maintaining oncologic efficacy. Laparoscopic radical gastric cancer surgery, including laparoscopic-assisted and total laparoscopic approaches, has been shown in numerous studies to offer reduced trauma and quicker recovery compared to traditional open radical surgery (31, 32). Especially the Da Vinci robotic surgical system has garnered significant attention in minimally invasive surgery, with a recent meta-analysis presenting compelling evidence supporting the efficacy of RG over OG for gastric cancer treatment. However no large-sample meta-analysis has been performed to compare the efficacy and safety of RG versus OG for GC, Especially the lack of evaluation of long-term outcomes.

This meta-analysis showed that the operation time in the OG group was longer than that in the OC group, which similar to the findings in the existing literature (33), primarily attributed to the intricate docking process of the robotic system. This process necessitates precise adjustments of the robot's position and angle to optimize the surgical field and operating space. In contrast to conventional open surgical instruments, robotic surgical instruments offer a more refined and distinct operational mode, requiring surgeons to undergo a requisite number of surgical procedures to attain proficiency. Kim et al. reported (34) that 40–50 surgeries were required to master the procedure. Despite the prolonged operation duration, the robotic group demonstrates notable benefits in reducing surgical blood loss and shortening hospital stay. The substantial decrease in clinical blood loss (−86.71 mL) underscores the exceptional precision of robotic surgery. The reduced hospital stay duration (−3.17 days) corroborates findings from analogous studies (35, 36), alleviating both the financial burden on patients and the risk of complications such as in-hospital infections. The integration of the Enhanced Recovery After Surgery (ERAS) concept in clinical practice further underscores the advantages of RG (37). ERAS emphasizes employing multimodal interventions to mitigate the surgical stress response and expedite patient recovery. The precision and minimally invasive nature of robotic surgery align closely with the ERAS concept, better addressing patients' requirements for swift recuperation. The enhanced 3D visualization, flexible instruments, and superior ergonomic design of RG are pivotal in achieving precise blood vessel and tissue space dissections (38). The augmented 3D vision system furnishes a stereoscopic and realistic surgical field, enabling surgeons to more accurately assess tissue distances and hierarchies. The flexible instruments replicate human wrist movements for intricate operations in confined surgical spaces, while the superior ergonomic design diminishes surgeon fatigue, enhancing surgical stability and accuracy.

In terms of oncological parameters, comparable lymph node resection numbers and similar positive lymph node counts suggest that RG is non-inferior to OG in lymph node dissection, aligning with findings from a multicenter RCT (39). This contrasts with a prior study by Chen reporting a lower number of positive lymph nodes in the RG cohort compared to the OG cohort. This discrepancy is likely attributed to the incorporation of a broader range of studies and a larger sample size in the present analysis, enhancing the objectivity and precision of the results. Enlarging the sample size enables a more comprehensive representation of diverse patient cohorts and surgical outcomes, thereby mitigating potential biases from individual studies. Notably, the higher R0 resection underscores the technical proficiency in achieving tumor-free margins, a critical prognostic factor in gastric cancer (40). Through careful evaluation of the included studies, we found that some studies (19, 22, 27) lacked randomization in case allocation, and that the RG group included a higher proportion of GC cases with lower surgical difficulty and less surrounding tissue invasion than the OG group. In addition, the RG group had a higher rate of preoperative chemotherapy. Preoperative chemotherapy can effectively reduce tumor size and control micrometastases, thereby creating more favorable conditions for complete surgical resection; this factor may also influence the R0 resection rate. Therefore, it has not yet been effectively demonstrated that robots have real advantages over open surgery in the surgical treatment of gastric cancer in terms of R0 resection rate. In the future, it is necessary to conduct a randomized controlled study by expanding the sample size, standardizing baseline indicators such as tumor stage and lesion location, and at the same time, combining data from multi-center studies to further verify the difference in R0 resection rates between robot and traditional laparoscopic or open surgery. Meanwhile, the 5-year survival rate equivalence (HR: −0.1; 95%) closely paralleled findings from a recent comparative study between RG and LG (41), suggesting that short-term patient benefits have no discernible impact on long-term tumor outcomes. This dispels prior doubts regarding the efficacy of minimally invasive surgery and underscores robotic-assisted surgery as a feasible option for resectable conditions.

Furthermore, in terms of safety assessment, the RG group exhibited a significant reduction in overall complications, highlighting the efficacy of robotic systems in minimizing tissue trauma. Robotic surgery's precision can mitigate inadvertent damage to adjacent tissues, thereby lowering postoperative complication rates. For instance, the precise manipulation of robotic tools during incisions and suturing can prevent harm to nearby nerves, blood vessels, and organs, consequently decreasing the likelihood of complications like bleeding, infection, and anastomotic leakage. Moreover, comparable postoperative mortality between the groups underscore the safety profile of robotic surgery.

This meta-analysis also has several limitations: 1) variations in surgical expertise across different centers may significantly impact the results. Surgeons at different centers exhibit varying proficiencies in robotic and open surgeries, alongside differences in surgical experience accumulation. Such discrepancies in surgical proficiency could introduce biases in the study outcomes across centers, thereby affecting the generalizability and accuracy of the results. 2) the absence of high-quality RCTs hinders the synthesis of conclusions akin to Level 1 evidence. This scarcity may stem from the high costs and stringent technical demands of robotic surgery, posing challenges in conducting large-scale RCTs. 3) the study encompasses a limited number of publications on long-term oncological outcomes with a restricted sample size. This limitation may be attributed to the prolonged survival period of gastric cancer patients, necessitating extensive time and resources for long-term follow-up studies, resulting in a scarcity of relevant literature.

## 5 Conclusions and clinical significance

RG represents a judicious choice for proficient medical centers specializing in robotic surgery, offering a favorable compromise between surgical invasiveness and oncological precision. Overcoming the learning curve, typically spanning 40–50 surgeries, poses a significant challenge. Enhanced outcomes and reduced operation duration can be achieved through centralized training programs and regular equipment upgrades. Subsequent investigations could center on long-term outcome and quality-of-life metrics to furnish clinicians with valuable insights for policymaking decisions.
