# Supplementary material for: The value of robot-assisted gastrectomy in the treatment of gastric cancer: a systematic review and meta-analysis
Source: Front Surg. 2026 Jun 9;13:1828368. doi: 10.3389/fsurg.2026.1828368 (PMC13286787; doi:10.3389/fsurg.2026.1828368)
Supplement: Supplementary file 1 [file Datasheet1.docx]

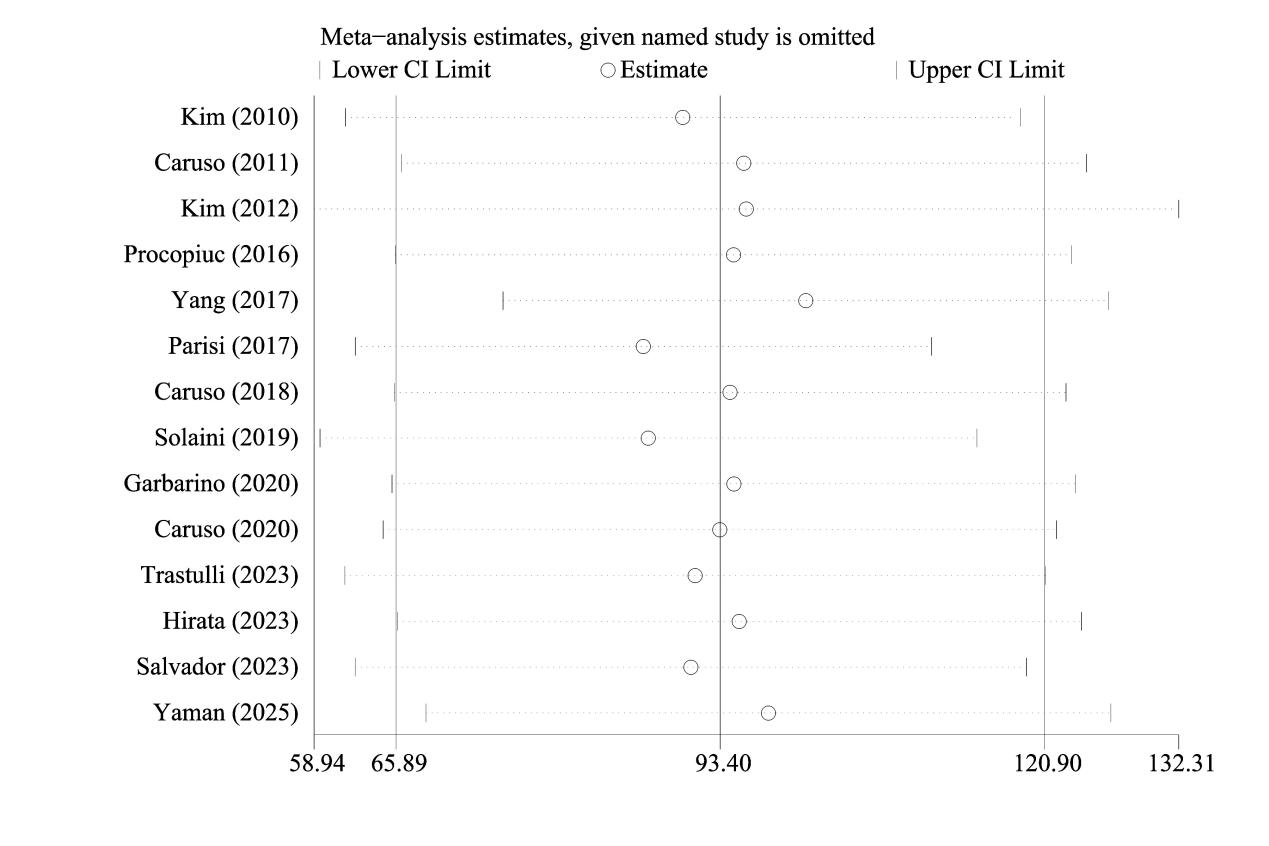


**Fig. S1.** The analysis of the individual impact of operative time.


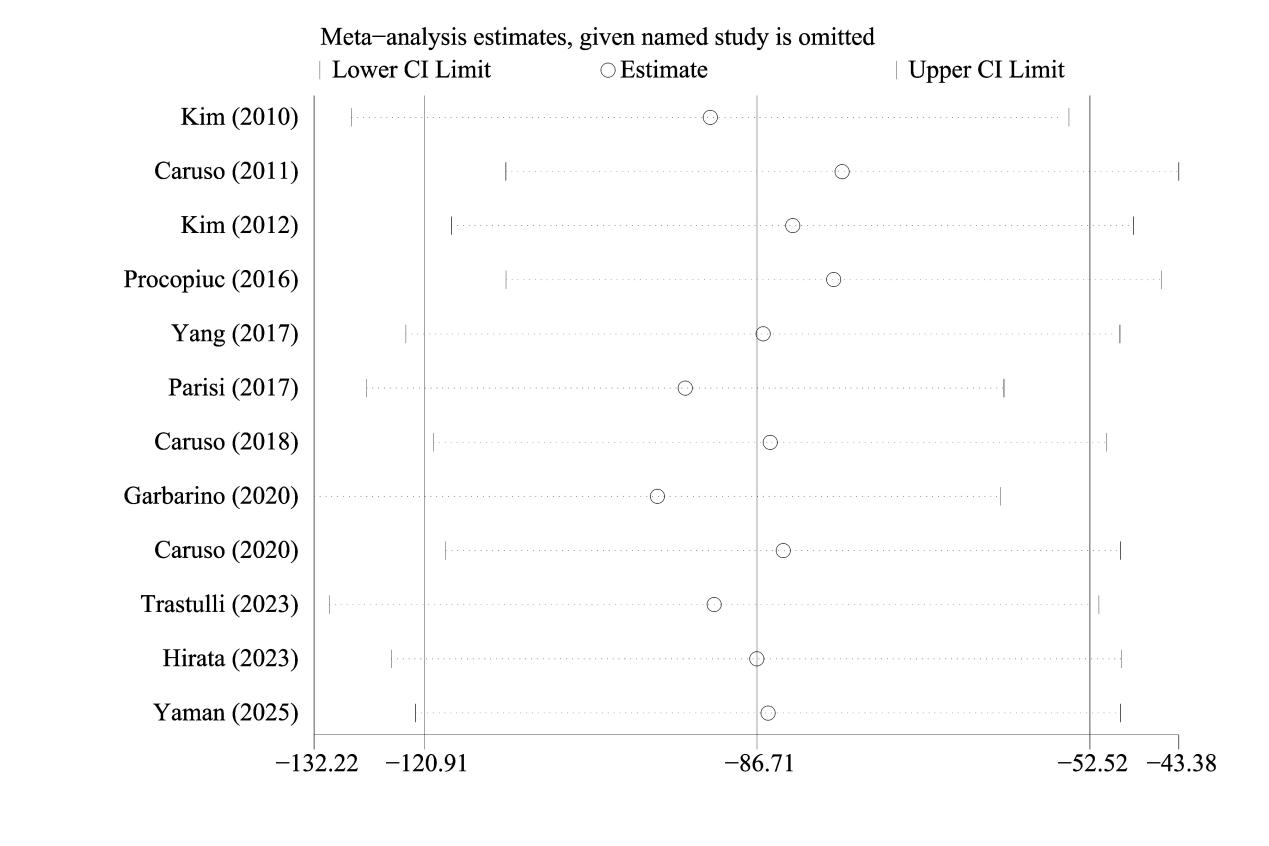


**Fig. S2.** The analysis of the individual impact of blood loss.


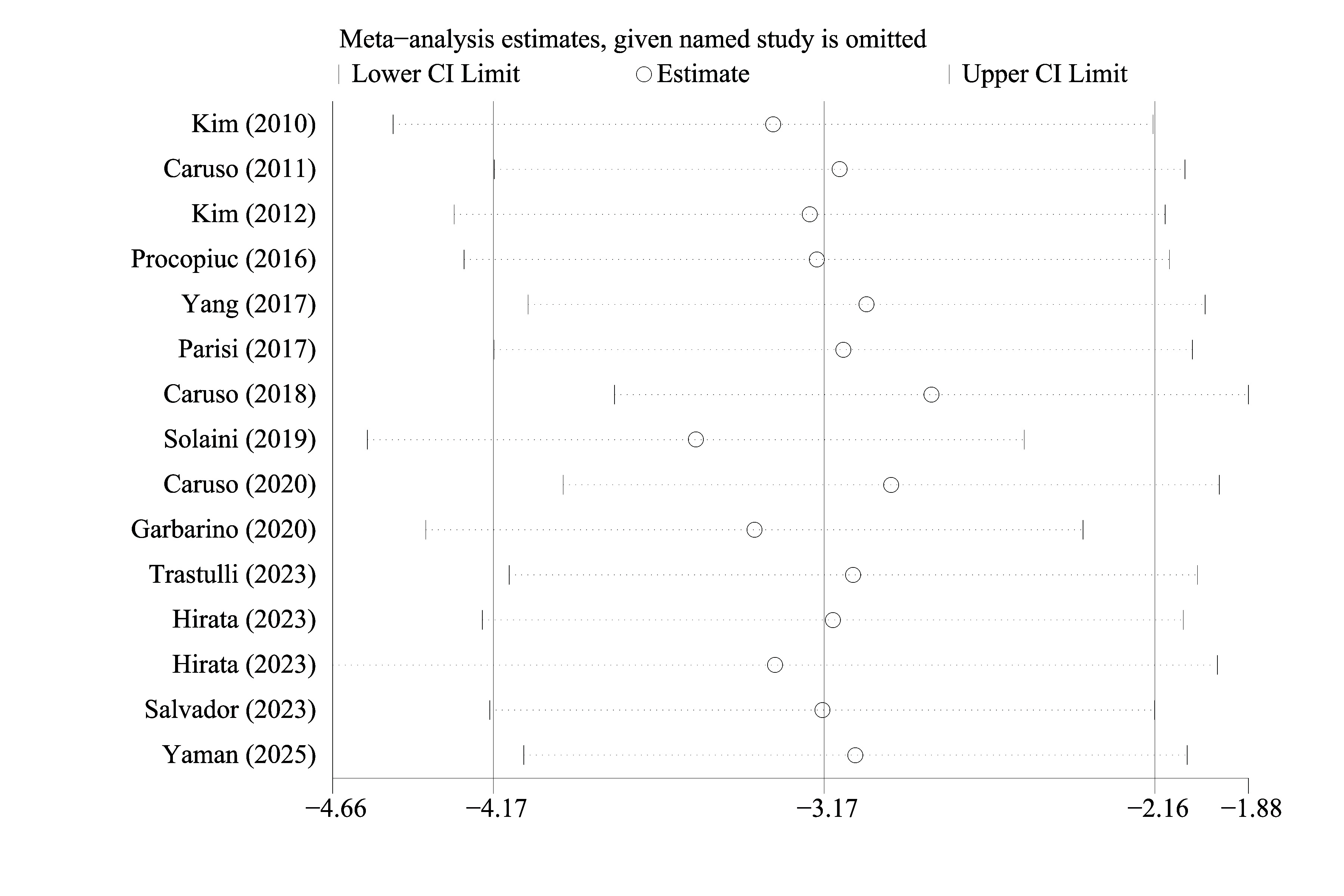


**Fig. S3.** The analysis of the individual impact of hospital stay.


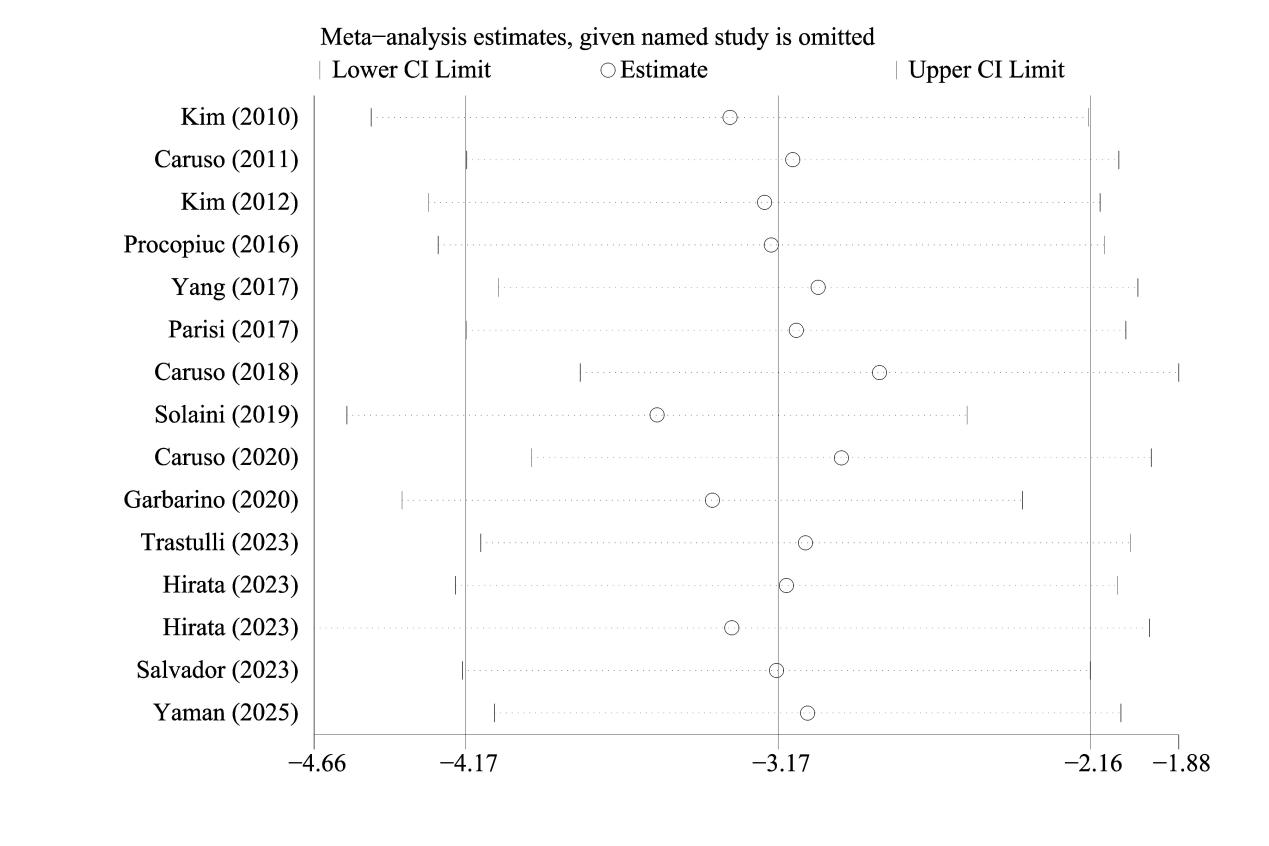


**Fig. S4.** The analysis of the individual impact of lymph nodes.


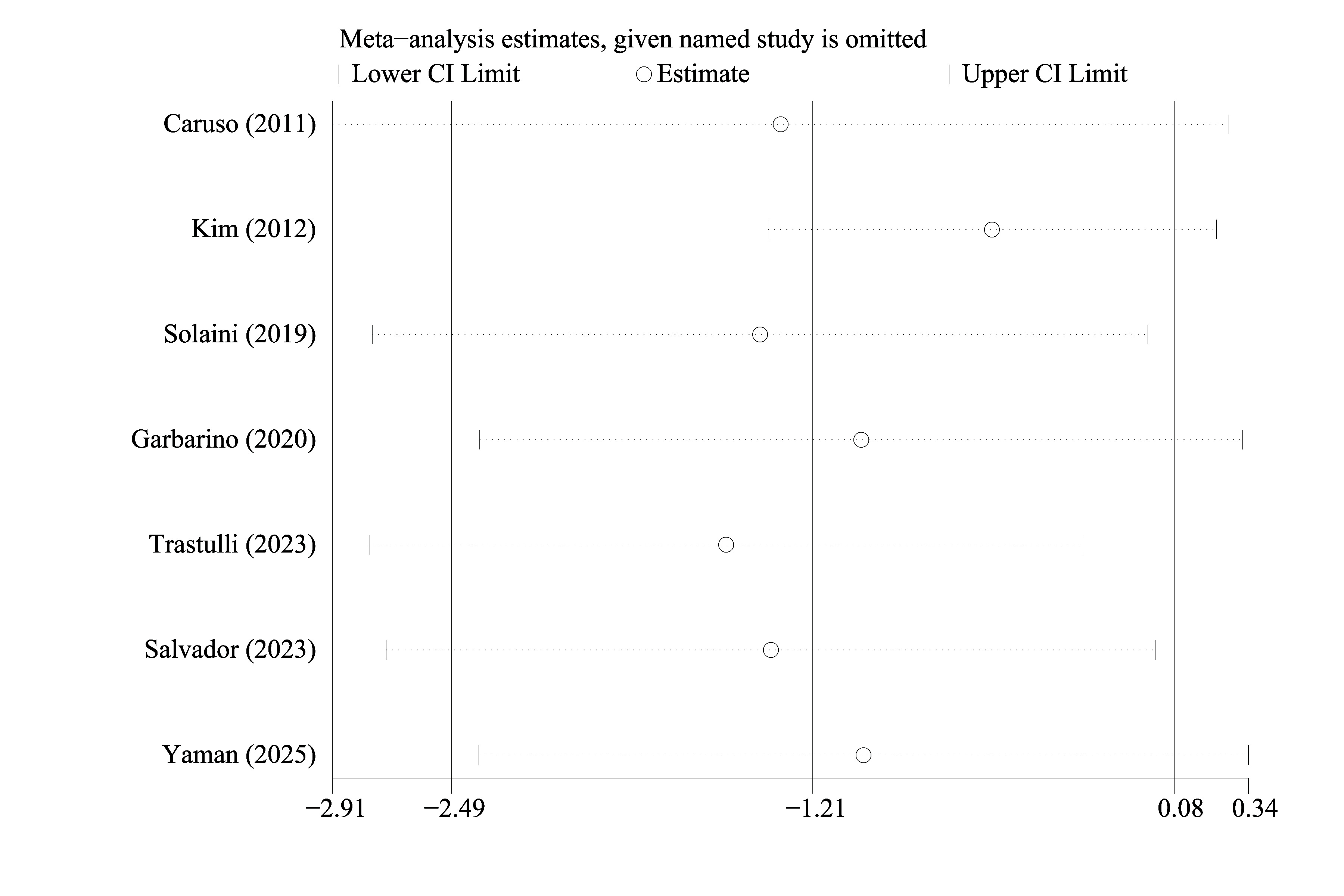


**Fig. S5.** The analysis of the individual impact of positive lymph nodes.


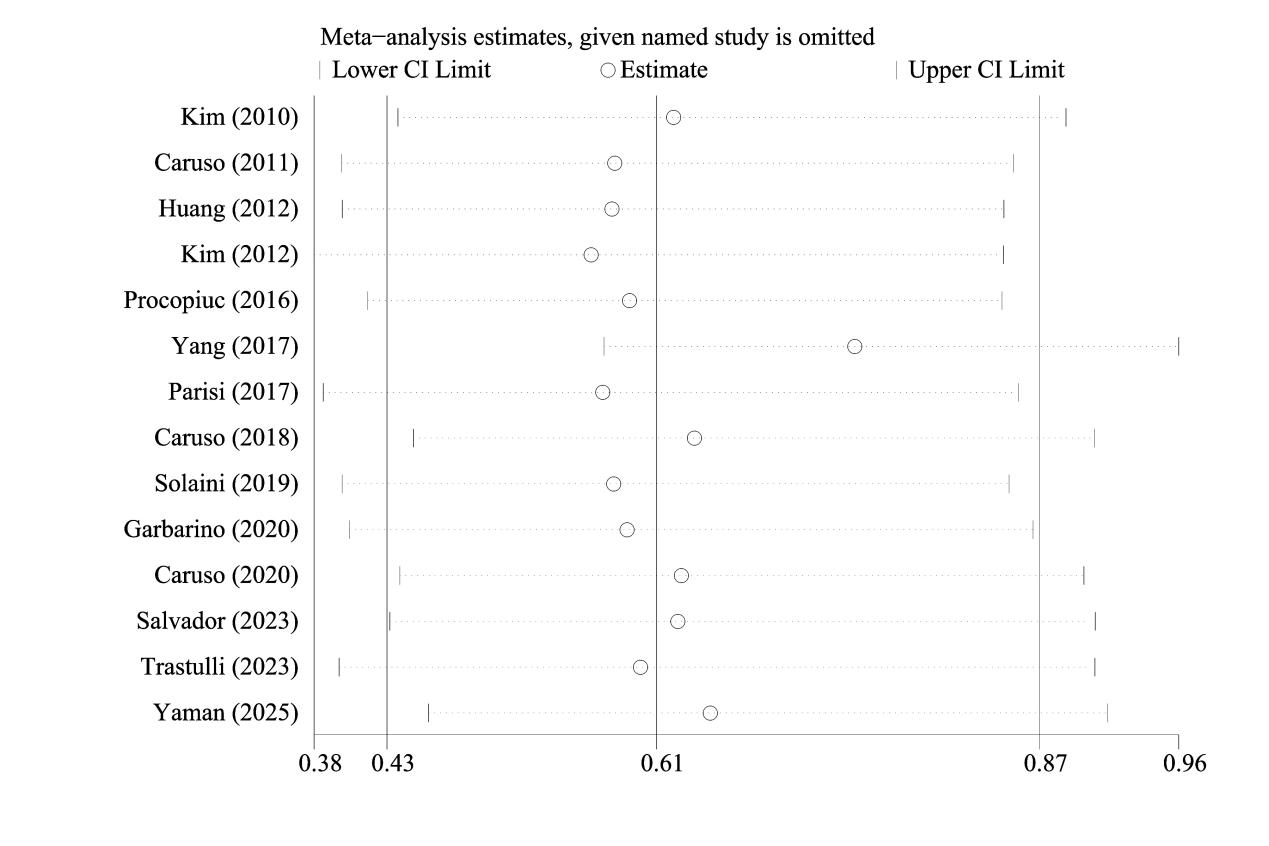


**Fig. S6.** The analysis of the individual impact of postoperative complication.
